# Cardiac concerns associated with strontium ranelate

**DOI:** 10.1517/14740338.2014.939169

**Published:** 2014-07-14

**Authors:** Jean-Yves Reginster

**Affiliations:** ^a^ University of Liège, Department of Public Health, Epidemiology and Health Economics, CHU Sart-Tilman, 4020 Liège, Belgium+32 4270 3257; +32 4270 3253; jyreginster@ulg.ac.be

**Keywords:** cardiac safety, myocardial infarction, osteoporosis, strontium ranelate

## Abstract

***Introduction:*** Strontium ranelate is proven to reduce vertebral and non-vertebral fracture risk in osteoporosis. Concerns about cardiac safety have led to a new contraindication to strontium ranelate in patients with uncontrolled hypertension and/or current or past history of ischaemic heart disease, peripheral arterial disease and/or cerebrovascular disease.

***Areas covered:*** A literature search was performed; data were also collected from the European Medicines Agency website. Randomised controlled trial (RCT) data indicate a higher incidence of non-adjudicated myocardial infarction (MI) with strontium ranelate versus placebo (1.7 vs 1.1%; odds ratio [OR]: 1.6; 95% CI: 1.07 – 2.38; p = 0.020) (Mantel-Haenzel estimate of the OR). There was no increase in cardiovascular mortality. MI risk was mitigated by excluding patients with cardiovascular contraindications (OR: 0.99; 95% CI: 0.48 – 2.04; p = 0.988). Three observational studies performed in the context of real-life medical practice in the UK and Denmark did not report a signal.

***Expert opinion:*** The increased risk for cardiac events with strontium ranelate has been detected in RCTs but not in real life. Excluding patients with cardiovascular contraindications appears to be an effective measure for controlling the risk of MI. Strontium ranelate remains a useful therapeutic alternative in patients with severe osteoporosis without cardiovascular contraindications who are unable to take another osteoporosis treatment.

## 1. Introduction

Strontium ranelate, an osteoporosis medication registered in Europe in 2004, has been studied in a range of randomised controlled trials (RCTs) [1-6]. It was originally indicated for the treatment of women with postmenopausal osteoporosis to reduce the risk for vertebral and hip fracture. The efficacy of strontium ranelate for preventing fracture in osteoporosis is well established, having been demonstrated in two pivotal RCTs – Spinal Osteoporosis Therapeutic Intervention (SOTI) trial and TReatment Of Peripheral OSteoporosis (TROPOS) [2,3]. SOTI showed that, over 3 years, treatment with strontium ranelate 2 g/day reduced the risk of vertebral fracture in postmenopausal osteoporotic women and increased lumbar spine bone mineral density [2]. Strontium ranelate was demonstrated to have an effect on non-vertebral fracture (including hip) in postmenopausal osteoporotic women in TROPOS [3]. Strontium ranelate also increases bone mineral density in osteoporotic men [4], and there is evidence that its antifracture efficacy is maintained up to 10 years [5,6].

The procedure for reviewing the safety of drugs in Europe changed in 2012 with the creation of the Pharmacovigilance Risk Assessment Committee at the European Medicines Agency (EMA) to regularly assess submitted periodic safety update reports (PSURs) [7]. In accordance with European regulations, a PSUR, containing the latest data from new RCTs of strontium ranelate, was submitted to the EMA. The PSUR noted a signal for increased incidence of myocardial infarction (MI) [8]. This analysis of cardiovascular data led the EMA to recommend a change in the indication of strontium ranelate, which is now restricted to patients with severe osteoporosis for whom treatment with other medicinal products is not possible. A new contraindication was also added in patients with uncontrolled hypertension and those with established, current or past history of ischaemic heart disease, peripheral arterial disease and/or cerebrovascular disease [1]. In this article, we review the evidence for these cardiac concerns from the RCTs, as well as the results from post-marketing surveillance and observational registry-based studies.

## 2. Methods

Relevant articles, reviews and abstracts were identified in a search of PubMed/MEDLINE and EMBASE for English-language articles published up to March 2014. The initial search strategy included the terms strontium ranelate, MI, ischaemia, ischaemic heart disease and cardiac safety, and yielded 13 items. Additional references were selected from appropriate reviews on strontium ranelate and the management of osteoporosis, as well as data available on the EMA website.

## 3. Evidence from RCTs for an effect on cardiac risk

The source of the cardiac concerns about strontium ranelate was the 13th PSUR submitted to the EMA in November 2012, which incorporated new safety data from additional clinical studies in male osteoporosis and osteoarthritis [4,9]. These data raised a new concern on cardiac safety, with a numerical imbalance in MI and led to a pooled analysis of RCT data from over 7000 postmenopausal women with osteoporosis (3803 on strontium ranelate and 3769 on placebo, who participated in two Phase II and five Phase III trials), who had similar baseline cardiovascular risk factors whether they were randomised to strontium ranelate or placebo [8]. The results indicated that non-adjudicated MI was more common with strontium ranelate than placebo (1.7 vs 1.1%; odds ratio [OR] [Mantel-Haenzel estimate of the OR]: 1.6; 95% CI: 1.07 – 2.38; p = 0.020) [8]. In this analysis, the estimated annual incidence of MI with strontium ranelate was 5.7 per 1000 patient-years versus 3.6 per 1000 patient-years with placebo.

There were no interactions with the majority of risk factors for MI, such as age, raised body mass index (> 25 kg/m^2^), diabetes, dyslipidaemia or smoking. The only significant interaction to be detected was with diastolic blood pressure (< 90 or ≥ 90 mmHg) (p = 0.014). Patients with elevated diastolic blood pressure ≥ 90 mmHg were at increased risk (OR: 3.49; 95% CI: 1.59 – 7.65), whereas those with diastolic blood pressure < 90 mm Hg had no increase in MI (OR: 1.11; 95% CI: 0.69 – 1.79). These data led to the new contraindication in patients with uncontrolled hypertension, the elimination of whom considerably reduces risk.

When patients with cardiovascular contraindications (n = 3142 [41%]) were excluded from the analysis, MI risk was also mitigated (OR: 0.99; 95% CI: 0.48 – 2.04; p = 0.988). When the analysis considered all ischaemic events, there was a similar incidence of events with strontium ranelate and placebo (3.9% in the strontium ranelate group versus 4.7% in the placebo group; OR: 0.82; 95% CI: 0.61 – 1.12). Furthermore, there was no increase in risk of cardiac-related mortality in this population, with similar incidences of cardiovascular death (OR: 0.96; 95% CI: 0.57 – 1.61) and sudden death (OR: 0.41; 95% CI: 0.14 – 1.17).

The potential impact of the new indication and contraindications on the antifracture efficacy of strontium ranelate was recently studied in a multivariate analysis from SOTI (826 patients on strontium ranelate, 814 patients on placebo) and TROPOS (2526 and 2503 patients, respectively) [10]. In the whole population of patients from SOTI and TROPOS, treatment with strontium ranelate was associated with a 20% (95% CI: 9 – 29%) decrease in osteoporotic clinical fractures and a 40% (95% CI: 31 – 48%) decrease in vertebral fractures assessed by semiquantitative morphometry. The new analysis used the primary data from the two studies and identified which patients had potential contraindications at baseline. In a parallel analysis, the impact of the restriction to patients with severe osteoporosis was also studied using two definitions of severity: the WHO criteria (a T-score of −2.5 or less and a prior fragility fracture) and the probability-based assessment of fracture risk (FRAX®). There was no significant interaction between treatment effect and the presence/absence of contraindications, or between treatment effect and severity (p > 0.30). In the absence of contraindications, there was a 16% (95% CI: 0 – 30%) reduction in risk for clinical osteoporotic fracture and a 36% (95% CI: 3 – 44%) reduction in risk for vertebral fracture. In patients with severe osteoporosis, risk reduction for clinical osteoporotic fractures was 24% (95% CI: 6 – 39%) using the WHO definition of severity and 26% (95% CI: 5 – 33%) using the FRAX definition of severity. The authors of this analysis concluded that the antifracture efficacy is maintained in patients with severe osteoporosis, and in whom strontium ranelate is not contraindicated [10].

## 4. Evidence from post-marketing surveillance and post-authorisation studies

No specific cardiac concerns were detected by regulatory post-marketing surveillance covering about 3.4 million patient-years of strontium ranelate treatment between September 2004 and September 2012 [8]. A 32-month observational prospective cohort study in 12,046 patients on strontium ranelate, which predates the PSUR, did not report any cardiac safety issues [11]. The mean age of this cohort was 69 years and nearly a third (30%) had hypertension and 16% had dyslipidaemia; rates of uncontrolled hypertension were not recorded. Annual incidence of MI was 1.3 per 1000 patient-years, with 33 cases of MI, acute coronary syndrome (ACS) or coronary artery occlusion. No case was considered treatment related [8].

## 5. Observational registry-based cardiac safety studies

Electronic healthcare records from the UK and Denmark have been analysed by three independent teams in three retrospective observational studies of the cardiac safety of strontium ranelate in osteoporosis [12-14]. A nested case-control study in the UK Clinical Practice Research Datalink (CPRD) [12], including 6487 patients receiving strontium ranelate (age 74.9 ± 11.5 years, cumulative exposure of ∼1 year), found no evidence for increased risk of cardiac events with strontium ranelate. Of the 112,445 women with osteoporosis in the CPRD, the incidence rate of first-definite MI was 3.24 per 1000 patient-years (95% CI: 3.07 – 3.41). There was no increase in risk of MI in postmenopausal osteoporotic women who were taking strontium ranelate (OR: 1.05; 95% CI: 0.68 – 1.61) or had previously taken strontium ranelate (OR: 1.12; 95% CI: 0.79 – 1.58) compared with those who had never taken strontium ranelate [12]. Similarly, there was no significant association of current or past use with the risk of hospitalisation with MI (OR: 0.84; 95 % CI: 0.54 – 1.30 and OR: 1.17; 95% CI: 0.83 – 1.66, respectively) or cardiovascular death (OR: 0.96; 95% CI: 0.76 – 1.21 and OR: 1.16; 95% CI: 0.94 – 1.43, respectively) compared with patients who had never taken strontium ranelate.

In a separate observational study, the Danish National Prescription Database was used to identify 3252 male and female patients aged > 50 years who commenced treatment with strontium ranelate between 2005 and 2007 and 35,606 users of other osteoporosis drugs (designated controls) [13]. Patients who had taken strontium ranelate were found to be older at baseline than controls (74.0 vs 71.8 years) and to have a higher prevalence of ischaemic diseases – prior MI (6.8 vs 6.4%), peripheral vascular disease (6.2 vs 5.3%) or cerebrovascular disease (11.3 vs 10.0%) [13]. The absolute incidence rates of MI were 13.3 per 1000 patient-years in women who used strontium ranelate versus 11.1 per 1000 patient-years in women using other osteoporotic medication. The corresponding absolute incidence rates of MI were higher in men (30.3 and 24.8 per 1000 patient-years). Risk of MI did not increase with strontium ranelate (men, hazard ratio (HR): 1.28; 95% CI: 0.74 – 2.20; women, HR: 1.05; 95% CI: 0.79 – 1.41), nor did the risk of fatal MI (men, HR: 0.50; 95% CI: 0.07 – 3.64; women, HR: 1.73; 95% CI: 0.84 – 3.53).

Another cohort study of Danish postmenopausal women explored whether treatment with strontium ranelate was related to increased risk of ACS (unstable angina or MI) [14]. All women 50 – 84 years of age and living in Denmark from January 2005 to December 2011 were eligible. There were 3252 patients treated with strontium ranelate who were compared with 35,606 users of other osteoporosis drugs (designated controls). The analysis failed to find a significant association between strontium ranelate and ACS. For new users, the incidence rates of ACS were 5.7 per 1000 patient-years for strontium ranelate and 6.3 per 1000 patient-years for users of alendronate or risedronate (HR: 0.89; 95% CI: 0.52 – 1.55) [14]. For patients switched from first-line bisphosphonate, the incidence rates of ACS were 9.9 per 1000 patient-years with strontium ranelate and 9.9 per 1000 patient-years with ibandronate (HR: 1.00; 95% CI: 0.49 – 2.05) [14].

## 6. Discussion

Data from pooled analysis of RCTs indicated that MI may be more common in patients receiving strontium ranelate than those receiving placebo and have led to new conditions of use for this agent. Elimination of the patients with the new contraindications from the data set mitigated the increased risk. The increased risk of MI with strontium ranelate treatment observed within the context of RCTs is not shown in studies exploring the use of strontium ranelate in the setting of daily clinical practice [15]. No signal has been observed for increased risk of MI with strontium ranelate in post-marketing pharmacovigilance, and a post-authorisation observational prospective cohort study in 12,046 patients on strontium ranelate gave no suggestion of an association [8,11]. Subsequent observational studies using electronic healthcare records did not report any increase in risk [12-14]. These observational data come with a number of associated limitations, such as underreporting and confounders, although every effort was made in the various studies to reduce such bias [11-14]. On the other hand, the clinical trial findings should be taken seriously with regard to the clinical use of this agent. In this context, the antifracture efficacy of strontium ranelate appears to be maintained in patients with severe osteoporosis and in those without the contraindications. The latest restrictions on the prescription of strontium ranelate recommended by the EMA, therefore, ensure that this treatment can continue to be prescribed safely to severe osteoporotic patients without contraindications, who cannot be treated by other osteoporosis medications, due to, for example, contraindications or intolerance, with appropriate follow-up by prescribers [16].

All osteoporosis treatments are associated with adverse effects, and some of these adverse effects may be cardiovascular in nature [17,18]. It is essential to take such effects into consideration in clinical practice, alongside the benefit of a treatment with regard to its risk for a specific type of patient. The practical steps of excluding patients with contraindicated ischaemic diseases from treatment with strontium ranelate is expected to mitigate the risk of cardiac ischaemic events, as will monitoring of cardiovascular risk every 6 – 12 months. To safeguard osteoporotic patients, proper cardiovascular follow-up is now a key to the continued use of strontium ranelate in osteoporosis. These risk minimisation measures are easy to apply and already part of good clinical practice.

## 7. Conclusion

The increased risk for cardiac events with strontium ranelate has been detected in randomised clinical trials but not in real-life observational studies. Excluding patients with cardiovascular contraindications from the analysis of RCTs appears to mitigate the increased MI risk with strontium ranelate. When other osteoporosis treatments cannot be used due to contraindications or intolerances, strontium ranelate remains an interesting alternative to decrease the risk of vertebral and non-vertebral fracture in osteoporosis.

## 8. Expert opinion

Quality information is fundamental to the accurate assessment of the benefits and risks of all medicines, including osteoporosis treatments. The EMA has improved the assessment of risk by enhancing the surveillance and reporting of side effects with the introduction of Good Pharmacovigilance Practices [7]. This formalisation of medicinal risk assessment now allows the EMA to examine the risks of medicines on a continuous basis and more comprehensively than in the past.

In the case of strontium ranelate, it has permitted the detection of a change in risk and the implementation of new measures for clinical practice. Although the data from RCTs indicated an increase in risk of MI, they also showed that elimination of patients with the new contraindications mitigated the risk. Further analysis of the trial data also demonstrated that the antifracture efficacy of strontium ranelate is not affected by the new contraindications in the management of severe osteoporosis. Rapid detection means that further analysis and safety measures can be carried out more quickly.

Three observational analyses carried out by separate groups in the setting of two different healthcare systems did not report a signal for increased cardiac risk with strontium ranelate treatment [12-14]. Why three observational studies failed to replicate the findings from a pooled analysis of data from RCTs remains a question for the research agenda. The observational studies had different designs and collected data from different types of sources but still came to the same conclusion. They were all highly robust, with sufficient statistical power. The results of sensitivity analyses were satisfactory. They all included a number of procedures to eliminate potential bias, although none managed to completely address the issue of a potential channelling bias, by which more fragile at-risk patients were receiving strontium ranelate. One possible explanation for the lack of a signal in these observational studies could be simply that the risk of MI with strontium ranelate is very low – and therefore more difficult to detect.

Strontium ranelate is an effective osteoporosis treatment in patients with severe osteoporosis and in those without the new contraindications [10,19]. There are patients who would benefit from the prescription of strontium ranelate as an alternative option, provided the contraindications are properly respected.

Article highlights.

- Strontium ranelate is an osteoporosis medication that reduces the risk of vertebral and hip fractures in men and women with osteoporosis, including those with severe osteoporosis.
- An analysis of randomised controlled trials of strontium ranelate found that non-adjudicated myocardial infarction was more common with strontium ranelate than placebo, but that cardiovascular mortality did not increase. Barring patients with cardiovascular contraindications from treatment with strontium ranelate from the analysis mitigated cardiac risk.
- Subsequent observational studies did not report a signal of increased cardiac risk with strontium ranelate.
- Strontium ranelate is now contraindicated in patients with uncontrolled hypertension and those with current or past history of ischaemic heart disease, peripheral arterial disease and/or cerebrovascular disease, and restricted to patients who cannot take other osteoporosis treatments.
- Thanks to regulatory measures to prevent cardiac vascular events, strontium ranelate remains an interesting option in osteoporosis.

This box summarises key points contained in the article.

## Declaration of interest

The author has received consulting fees or been paid for participation on advisory boards for Servier, Novartis, Negma, Lilly, Wyeth, Amgen, GlaxoSmithKline, Roche, Merckle, Nycomed-Takeda, NPS, IBSA-Genevrier, Theramex, UCB, Asahi Kasei and Endocyte. The author has received lecture fees when speaking at the invitation of a commercial sponsor from Servier, Merck Sharp and Dohme, Lilly, Rottapharm, IBSA, Genevrier, Novartis, Roche, GlaxoSmithKline, Merckle, Teijin, Teva, Analis, Theramex, Nycomed, NovoNordisk, Ebewee Pharma, Zodiac, Danone, Will Pharma and Amgen. Finally, the author has received grant support from Servier, Bristol Myers Squibb, Merck Sharp & Dohme, Rottapharm, Teva, Roche, Amgen, Lilly, Novartis, GlaxoSmithKline, Pfizer, Theramex, Danone, Organon, Therabel, Boehringer, Chiltern and Galapagos. The author has no other relevant affiliations or financial involvement with any organisation or entity with a financial interest in or financial conflict with the subject matter or materials discussed in the manuscript apart from those disclosed.
